# Syntaxin-3 is dispensable for basal neurotransmission and synaptic plasticity in postsynaptic hippocampal CA1 neurons

**DOI:** 10.1038/s41598-019-57388-6

**Published:** 2020-01-20

**Authors:** Shan Shi, Ke Ma, Na-Ryum Bin, Hidekiyo Harada, Xiaoyu Xie, Mengjia Huang, Haiyu Liu, Soomin Lee, Xue Fan Wang, Roberto Adachi, Philippe P. Monnier, Liang Zhang, Shuzo Sugita

**Affiliations:** 1 Department of Pediatrics, The First Hospital of Jilin University, Changchun, 130021 China; 2 Division of Fundamental Neurobiology, Krembil Research Institute, University Health Network, Toronto, Ontario M5T 2S8 Canada; 3 Department of Physiology, University of Toronto, Toronto, Ontario M5S 1A8 Canada; 4 Division of Genetics and Development, Krembil Research Institute, University Health Network, Ontario, M5T 2S8 Canada; 5 Department of Anesthesiology, Dalian Medical University, Dalian, Liaoning 116044 China; 6 Department of Neurosurgery, The First Hospital of Jilin University, Changchun, 130021 China; 7 Department of Pulmonary Medicine, The University of Texas MD Anderson Cancer Center, Houston, Texas 77030 USA; 8 Department of Ophthalmology & Vision Sciences, University of Toronto, Toronto, Ontario M5S 1A8 Canada; 9 Department of Medicine, Faculty of Medicine, University of Toronto, Toronto, Ontario M5S 1A8 Canada

**Keywords:** Spatial memory, Cellular neuroscience

## Abstract

Recent evidence suggests that SNARE fusion machinery play critical roles in postsynaptic neurotransmitter receptor trafficking, which is essential for synaptic plasticity. However, the key SNAREs involved remain highly controversial; syntaxin-3 and syntaxin-4 are leading candidates for the syntaxin isoform underlying postsynaptic plasticity. In a previous study, we showed that pyramidal-neuron specific conditional knockout (cKO) of syntaxin-4 significantly reduces basal transmission, synaptic plasticity and impairs postsynaptic receptor trafficking. However, this does not exclude a role for syntaxin-3 in such processes. Here, we generated and analyzed syntaxin-3 cKO mice. Extracellular field recordings in hippocampal slices showed that syntaxin-3 cKO did not exhibit significant changes in CA1 basal neurotransmission or in paired-pulse ratios. Importantly, there were no observed differences during LTP in comparison to control mice. Syntaxin-3 cKO mice performed similarly as the controls in spatial and contextual learning tasks. Consistent with the minimal effects of syntaxin-3 cKO, syntaxin-3 mRNA level was very low in hippocampal and cortex pyramidal neurons, but strongly expressed in the corpus callosum and caudate axon fibers. Together, our data suggest that syntaxin-3 is dispensable for hippocampal basal neurotransmission and synaptic plasticity, and further supports the notion that syntaxin-4 is the major isoform mediating these processes.

## Introduction

Synaptic transmission is essential for neuronal communication in the brain. During synaptic transmission, presynaptic neurons release neurotransmitters that bind to their respective receptors on the postsynaptic membrane. Ionotropic glutamate receptors (AMPARs and NMDARs) and GABA receptors on the postsynaptic membrane undergo receptor recycling. Receptor recycling is an essential process in synaptic plasticity such as long-term potentiation (LTP). LTP is a cellular correlate for higher-level cognitive functions of learning and memory^1–5^ and requires rapid modifications in the quantity and composition of postsynaptic glutamate receptors^4,6,7^. Despite its importance, the underlying mechanisms of postsynaptic membrane receptor trafficking still remain unclear.

Postsynaptic receptor trafficking employs a unique soluble NSF-attachment protein receptor (SNARE) complex that mediates the attachment and fusion of vesicles containing postsynaptic receptors to target membranes. The SNARE complex is composed of one vesicle membrane protein (v-SNARE; synaptobrevin) and two target membrane proteins (t-SNAREs; syntaxin and SNAP-25 isoforms)^8^. Botulinum neurotoxin B (BoNT/B) proteolyzes synaptobrevin-2 and when injected into CA1 pyramidal cells, blocks LTP induction^9^. This suggests that this v-SNARE is imperative for AMPAR delivery to the postsynaptic membrane during LTP. However, the particular isoforms of t-SNAREs involved in postsynaptic neuronal vesicle fusion remains controversial^10–13^.

A previous study using non-functional recombinant syntaxin-4 revealed that syntaxin-4 mediates activity-dependent AMPAR trafficking to synapses during LTP^14^, whereas another study utilizing syntaxin-3 knockdown (KD) demonstrated that syntaxin-3 is pivotal for the delivery of AMPARs to postsynaptic membranes during LTP, but not syntaxin-4^11^. Therefore, it appears that both syntaxin-3 and syntaxin-4 are potential t-SNAREs that mediate postsynaptic AMPAR delivery during LTP. Conversely, syntaxin-3 and syntaxin-4 KD had no effects on basal transmission, which suggests for the potential involvement of another syntaxin isoform in AMPAR delivery^11,14^.

In a previous study, we utilized live murine models to examine the role of syntaxin-4 in postsynaptic neurons by generating a pyramidal neuron-specific conditional knockout (cKO) for syntaxin-4^15,16^. Analysis of syntaxin-4 cKO revealed significant decreases in basal synaptic transmission, LTP, surface expression of both AMPARs and NMDARs, and impaired spatial learning. However, syntaxin-4 cKO also caused a drastic decrease in NMDA current which could impair NMDA- and Ca^2+^-dependent LTP induction^17,18^. Therefore, these data do not directly indicate a functional role of syntaxin-4 in AMPAR delivery during LTP and implicate a possible role of another syntaxin isoform, potentially syntaxin-3 in such processes^11^. In this aspect, it is important to analyze the role of syntaxin-3 in a similar manner to the previous analysis of syntaxin-4^15,16^. In this study, we generated syntaxin-3 cKO mice and performed electrophysiological and behavioral analyses to further examine the role of syntaxin-3 in postsynaptic basal neurotransmission, synaptic plasticity, learning and memory.

## Results

### Generation of forebrain-specific syntaxin-3 cKO mice

**Figure 1:**
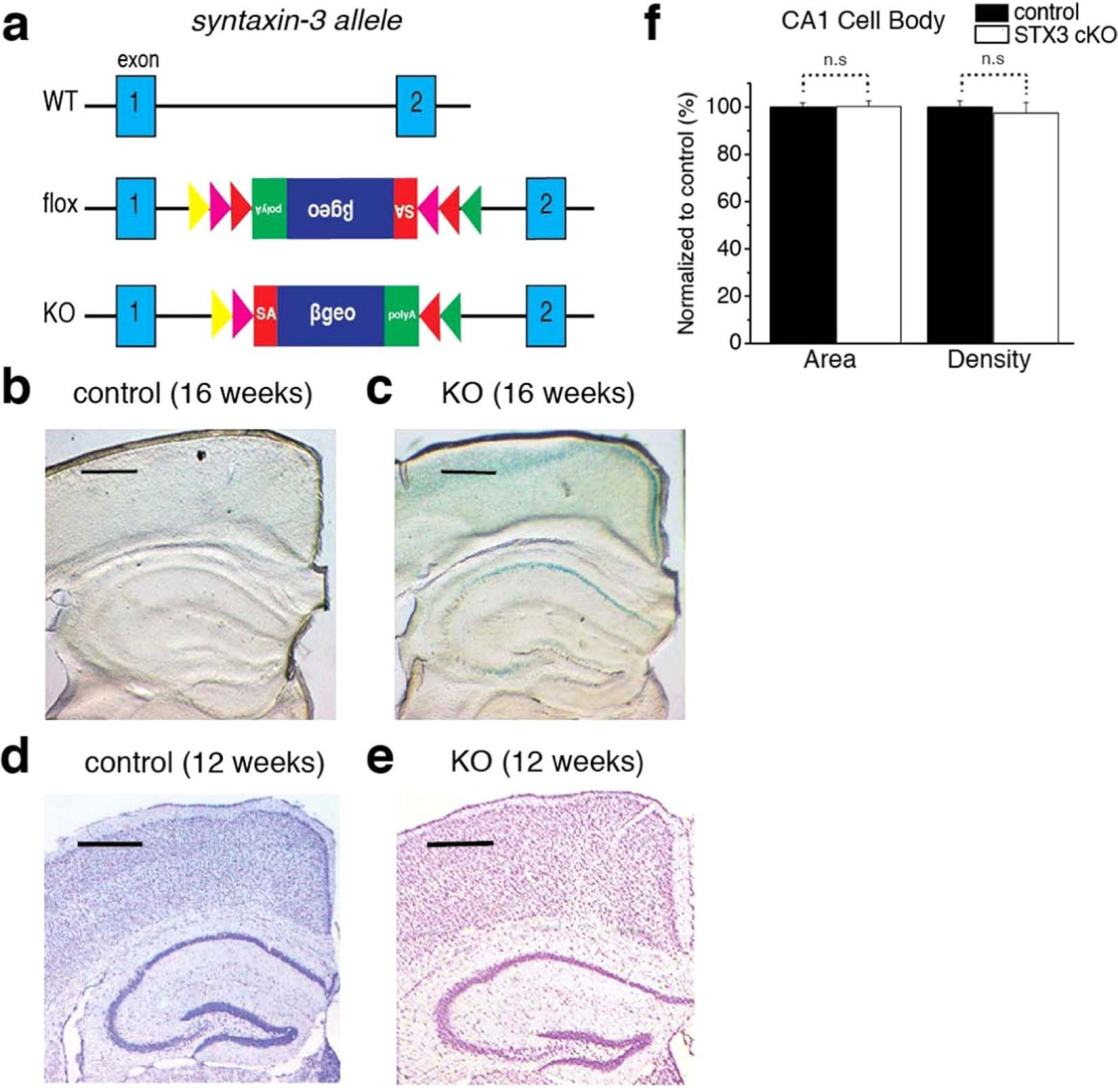
Generation of tissue-specific syntaxin-3 conditional KO mouse and electrophysiological and morphological analysis of the conditional KO mouse. (**a**) Schematic diagram illustrating the strategy used to obtain of syntaxin-3 conditional KO mice. Triangles, location and direction of recombination sites; yellow triangle, FRT; green triangle, F3; red triangle, loxP; pink triangle, lox511; red rectangle, splice acceptor (SA) site; βgeo, β-galactosidase/neomycin phosphotransferase fusion gene; green rectangle, polyadenylation (poly A) site. (**b**,**c**) X-Gal staining to examine the expression of β-galactosidase/neomycin fusion protein in control flox (**b**) and syntaxin-3 cKO (**c**) mice. (**d**,**e**) Nissl staining of CA1 cell body depicting the cell body area and density for control (**d**) and cKO (**e**) mice. Scale bar: 0.5 mm. (**f**) Quantification of CA1 cell body area and density. Using ImageJ (NIH, Bethesda, Maryland), the morphological CA1 cell body layer was manually selected, and the area and intensity (n = 22 for control, n = 35 for cKO) were measured. The two parameters were then normalized to the respective control. N.S. indicates nonsignificant (p > 0.05) in independent two-sample t-test.

Since global knockout of syntaxin-3 in mice leads to embryonic lethality^19^, we generated pyramidal neuron specific KO of syntaxin-3. We used syntaxin-3 flox mice that were previously successfully utilized in generating a mast cell specific syntaxin-3 cKO^19^. To eliminate syntaxin-3 expression from CA1 pyramidal neurons, we crossed syntaxin-3 flox/flox mice with mice expressing CaMK2a-Cre (Jacksons Lab) where Cre expression is distinctively strong in CA1 pyramidal neurons of the hippocampus^20,21^. The KO allele was created through gene trapping in which Cre recombinase induces the inversion of the gene trap to its sense orientation, ceasing syntaxin-3 expression and inducing the expression of a reporter gene, namely β-galactosidase/neomycin phosphotransferase fusion (β-geo) gene (Fig. 1a). Previously, the use of gene trapping in this manner had successfully eliminated the expression of syntaxin-3 in mast cells which resulted in significant impairment of exocytosis^19^.

Although Cre recombinase driven by the CaMK2a promoter has been demonstrated to be highly specific to the CA1 pyramidal layer in 8–10 week old animals^20,21^, its expression can be extended to other hippocampal areas such as the CA3 and dentate gyrus throughout development^22,23^. To examine the region specificity of the knockout, we performed X-gal staining to detect β-galactosidase activity in brain slices from syntaxin-3 cKO and control flox mice to confirm the generation of the syntaxin-3 KO allele. At 16 weeks, staining signals in CA1 neurons were clearly observed in cKO mice but not in control flox mice (Fig. 1b,c). However, the staining in cKO mice was not very specific to CA1 neurons as it was also observed in the CA3 region and the cortex (Fig. 1c). To examine whether a more region-specific staining could be observed at earlier time points, we performed the staining at different ages (8, 10, 12 weeks) (see Supplementary Fig. S1). To our surprise, X-gal staining was barely detected at 8 weeks, suggesting that Cre-dependent deletion had not occurred by this age (see Supplementary Fig. S1). In contrast, staining in CA1 neurons was clearly observed at 10 and 12 weeks (see Supplementary Fig. S1). However, the staining was still not very specific to CA1 neurons. Together, these results suggest that syntaxin-3 removal in cKO mice does not exclusively occur in CA1 neurons but also in other pyramidal neurons even at 10–12 weeks old. Therefore, we need to consider both presynaptic (i.e., CA3 neurons) and postsynaptic (i.e., CA1 neurons) factors when examining CA3-CA1 synaptic phenotypes in our syntaxin-3 cKO mice. To examine the function of syntaxin-3, we used age matched mice that ranged between 10–24 weeks old for analysis. However, the majority of mice that were analyzed in this study were ~12 weeks old.

We examined whether the removal of syntaxin-3 in CA1 pyramidal neurons induced gross histological changes when compared to the control by Nissl staining. No significant differences using independent two-sample t-test in cell area (control: n = 22 slices, cKO: n = 35 slices, t(55) = -0.05, p = 0.96) and density (control: n = 22, cKO: n = 35, t(55) = 0.437, p = 0.664) were detected between the cKO and control (Fig. 1d–f), which concludes that syntaxin-3 deletion from pyramidal neurons does not significantly affect the gross morphology of the hippocampus.

### Low syntaxin-3 mRNA and protein expression in hippocampal and cortex pyramidal neurons

**Figure 2:**
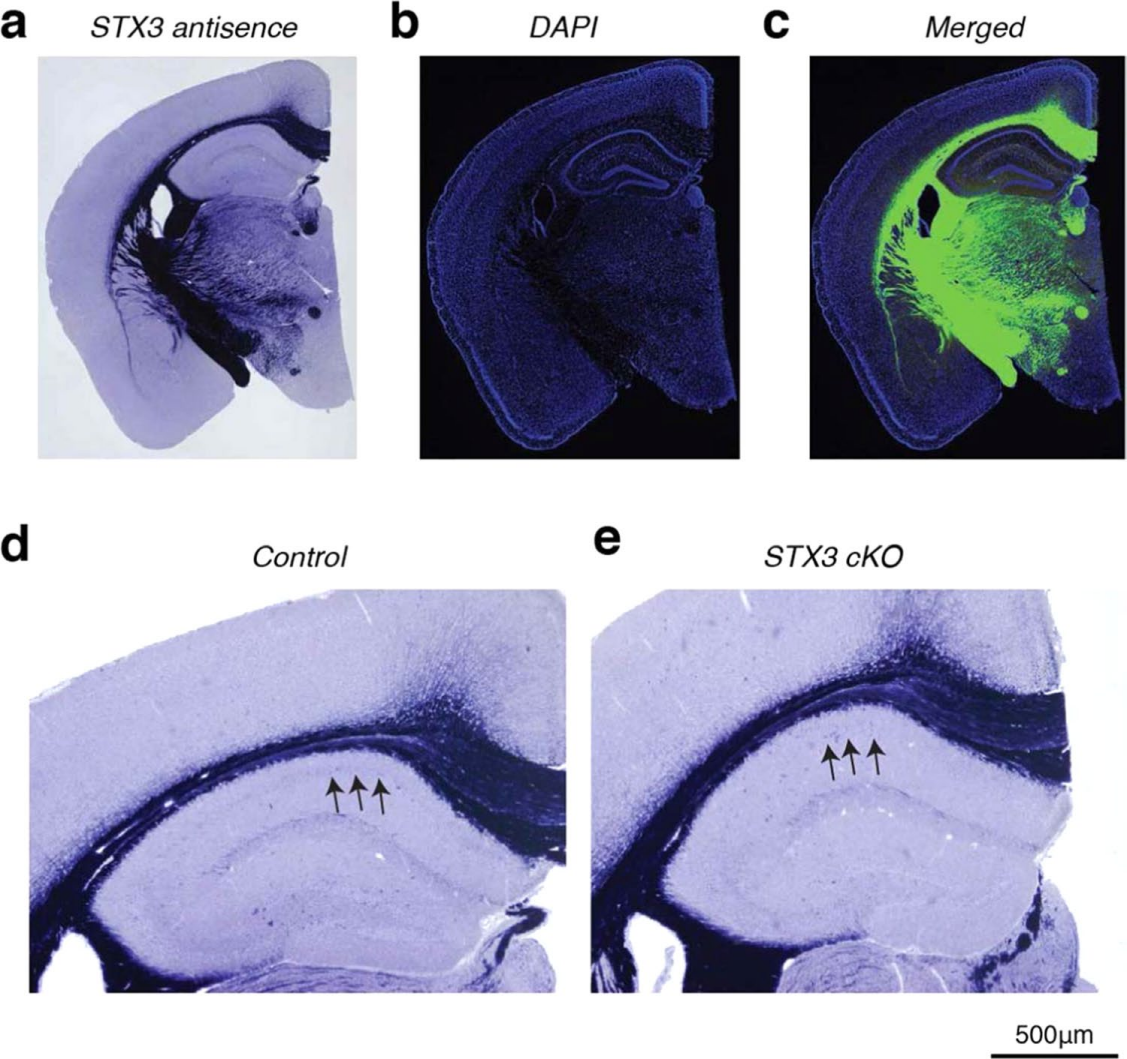
Endogenous syntaxin-3 mRNA expression in CA1 neurons is very low and dissipates in syntaxin-3 cKO mice. (**a**-**c**) *In situ* hybridization using syntaxin-3 3′ half-antisense probe (**a**), DAPI staining (**b**), and merged signals (**c**) in control brain slice. Antisense staining in (**c)** was shown in green. (**d**,**e**) Higher magnification of the hippocampal region in control (**d**) and cKO (**e**) brain slices. Arrows indicate the area of CA1 neurons. Scale: 500 µm.

To confirm the knockout of syntaxin-3 in syntaxin-3 cKO mice, we examined endogenous syntaxin-3 mRNA expression levels in the brain. Using *in situ* hybridization we determined the locations of syntaxin-3 mRNA expression. Differences in mRNA expression levels of hippocampal and cortex pyramidal neurons were measured between control and syntaxin-3 cKO mice (Fig. 2). Since transcription of syntaxin-3 is trapped after exon 1 in cKO mice (Fig. 1a), we generated an antisense probe of ~500 bp cDNA consisting of the 3′ portion (from exons 6–11) of mouse syntaxin-3. Thus, using this 3′ half-probe, we could observe differences in syntaxin-3 mRNA expression between the control and cKO groups. We found that in both groups, the probe strongly stained the corpus callosum and caudate axon fibers. Unexpectedly, we barely detected a signal in the hippocampus and cortex in both groups (Fig. 2a–c) suggesting low endogenous levels of syntaxin-3. After long exposure, we detected a very weak (yet still almost undetectable) signals in the CA1 neurons (indicated by arrows) of the control slice (Fig. 2d), which was not present in the cKO slice (Fig. 2e), confirming successful gene trapping in pyramidal neurons. These results suggest that the syntaxin-3 mRNA expression in the hippocampus and cortex is unexpectedly low but further confirm the effectiveness of gene trapping in impeding syntaxin-3 expression in cKO mice.

To examine whether the protein level of syntaxin-3 corresponds with the mRNA level in CA1 neurons, we performed immunofluorescence microscopy of the hippocampus. We used a polyclonal antibody that has been successfully used in previous studies^24–26^ to detect syntaxin-3 in mouse photoreceptor cells. This antibody failed to detect syntaxin-3 in the CA1 neurons (Supplementary Fig. S2), confirming that the expression level of syntaxin-3 is indeed low in CA1 neurons.

### Basal CA1 transmission is maintained in syntaxin-3 cKO mice

**Figure 3:**
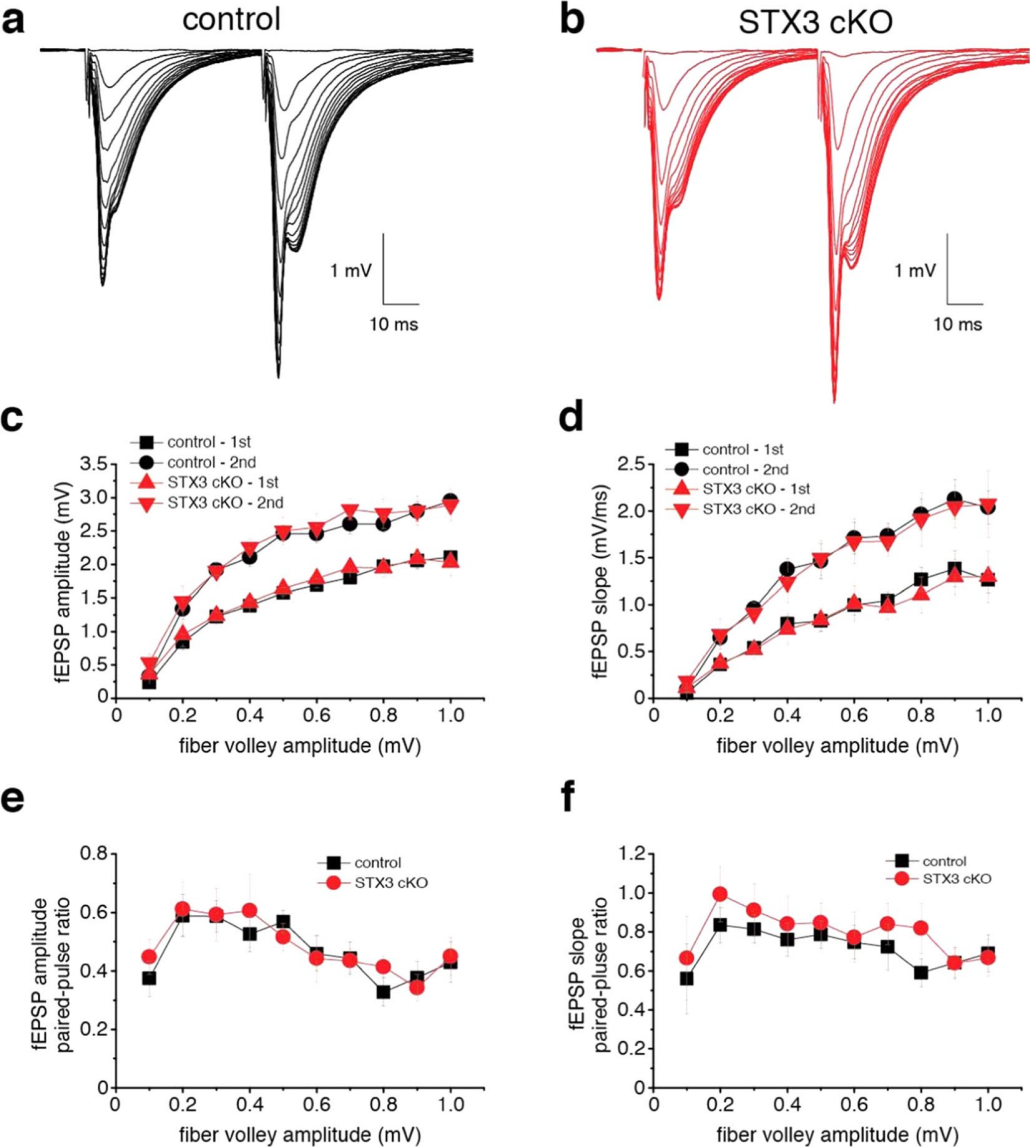
Tissue-specific syntaxin-3 conditional KO mice do not decrease in basal synaptic transmission. Schaffer collateral axonal fibers were administered two successive stimulations from 10 to 150 µA and the resulting local fEPSPs from apical dendrites of CA1 pyramidal neurons were recorded. (**a**,**b**) Averaged traces of dendritic fEPSPs from syntaxin-3 flox/flox (control) (**a**) and syntaxin-3 flox/flox; CaMK2a-Cre (cKO) (**b**). (C and D) fEPSP amplitudes (**c**) or slopes (**d**) were plotted against presynaptic fiber volley amplitudes. (E and F) Paired-pulse ratios of amplitudes (**e**) or slopes (**f**) were plotted against presynaptic fiber volley amplitudes. Error bars indicate SEM (animal: n = 16 for control and n = 11 for syntaxin-3 cKO groups).

To determine whether syntaxin-3 cKO from pyramidal neurons induce changes in basal neurotransmission, we prepared acute hippocampal slices and recorded field excitatory postsynaptic potential (fEPSP) from apical dendrites of CA1 pyramidal neurons (Fig. 3). Increasing the stimulation intensity increased the number of presynaptic axonal recruitments, which was quantified by fiber volley amplitudes. We used the fiber volley amplitudes to represent the “input” to generate input-output curves for comparisons of fEPSP amplitudes and slopes between the control and syntaxin-3 cKO groups (Fig. 3c,d). As the fiber volley amplitudes increased, fEPSP amplitudes and slopes from CA1 neurons elevated in both the control and syntaxin-3 cKO groups (Fig. 3a–d). Additionally, we observed that the fEPSP amplitudes and slopes were similar between the syntaxin-3 cKO mice and control mice (Fig. 3c–f). To determine the effects of syntaxin-3 deletion on presynaptic glutamate release, we gave two successive stimulations separated by 50 ms to measure the paired-pulse ratio between the second and first response (Fig. 3a,b,e,f). The paired-pulse ratio in the syntaxin-3 cKO remained similar to the control (Fig. 3e,f) which indicates that presynaptic release from syntaxin-3 cKO and control floxed neurons are similar. Collectively, our results suggest that deletion of syntaxin-3 leads to insignificant changes in basal synaptic transmission.

### CA1 long-term potentiation is preserved in syntaxin-3 cKO mice

**Figure 4:**
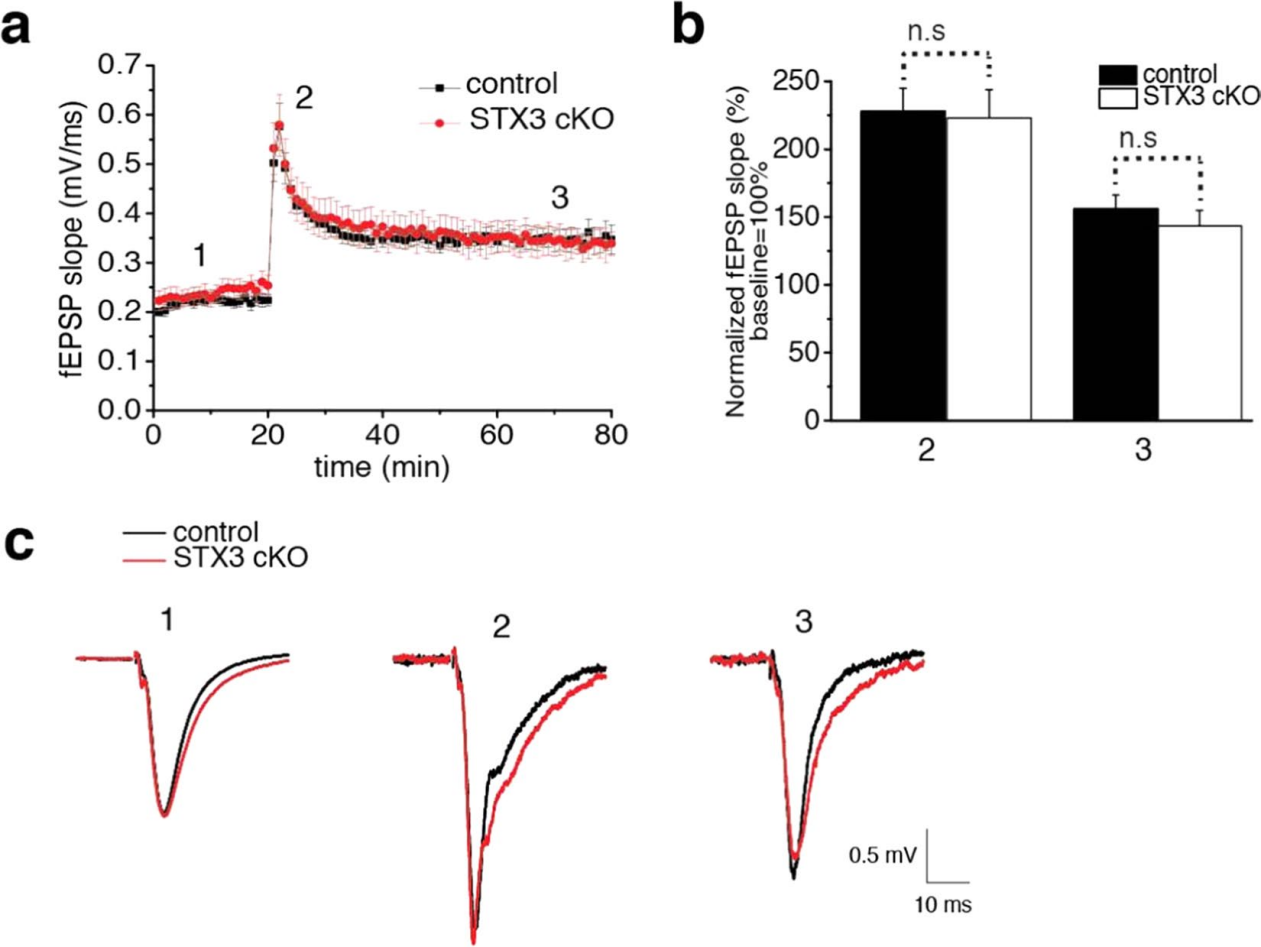
Syntaxin-3 cKO exbibits normal long-term potentiation. (**a,b**) Average fEPSPs slope (**a**) in control and syntaxin-3 cKO slices (**b**) 20 min before (1), immediately after theta burst stimulation (15 bursts of 4 pulses at 100 Hz with interburst internal of 200 msec) (2), and 60 min after (3). Throughout recording, the stimulation intensity was set to give a baseline fEPSP slope 30% of maximum evoked slopes. Error bars indicate SEM (animals: n = 10 for control and 11 for syntaxin-3 cKO). N.S. indicates nonsignificant differences between the two groups (p > 0.05); two-sample t-test. (**c**) Representative recordings of fEPSPs of control and syntaxin-3 cKO at baseline (1), immediately after LTP induction (2) and 50 min after LTP induction (3).

Previous studies have shown that postsynaptic SNARE proteins are crucial for the activity-dependent trafficking of ionotropic glutamate receptors during synaptic plasticity such as LTP^9,11,14^. Furthermore, the knockdown of syntaxin-3 impairs synaptic plasticity as it diminishes AMPAR delivery to synapses during LTP without affecting basal transmission^11^. Therefore, we investigated whether a conditional deletion of syntaxin-3 in postsynaptic CA1 neurons could also result in perturbation of LTP. For this purpose, we used acutely prepared hippocampal slices and measured the apical dendritic fEPSP from the CA1 before and after LTP induction via theta-burst stimulation in Schaffer collateral axons (Fig. 4). The magnitudes of post-tetanic potentiation (PTP) and later maintenance phase of LTP were compared between the control and syntaxin-3 cKO groups (Fig. 4). Immediately after delivering theta-burst stimulation, LTP was induced as PTP responses were more than 200% of the baseline in the control group (Fig. 4a–c). PTP responses then transitioned into LTP maintenance phases where the responses were stabilized to give ~150% of the baseline that lasted as long as 60 minutes after the theta-burst stimulation (Fig. 4a–c). To our surprise, the induction and maintenance of LTP were also similarly observed in the syntaxin-3 cKO group. (Fig. 4a–c). Independent two-sample t-test showed no significant differences between two groups (slope at immediately (shown as 2) after LTP induction, control group: 228 ± 17% of base line, n = 10, cKO group: 223 ± 21%, n = 11, t(19) = 0.19, p = 0.85; slope at 50 min (shown as 3) after LTP induction, control group: 156 ± 10%, n = 10, cKO group: n = 11, t(19) = 0.83, p = 0.42) Therefore, these results indicate that syntaxin-3 is dispensable for CA1 LTP induction and maintenance.

### Syntaxin-3 cKO mice do not exhibit impaired learning

**Figure 5:**
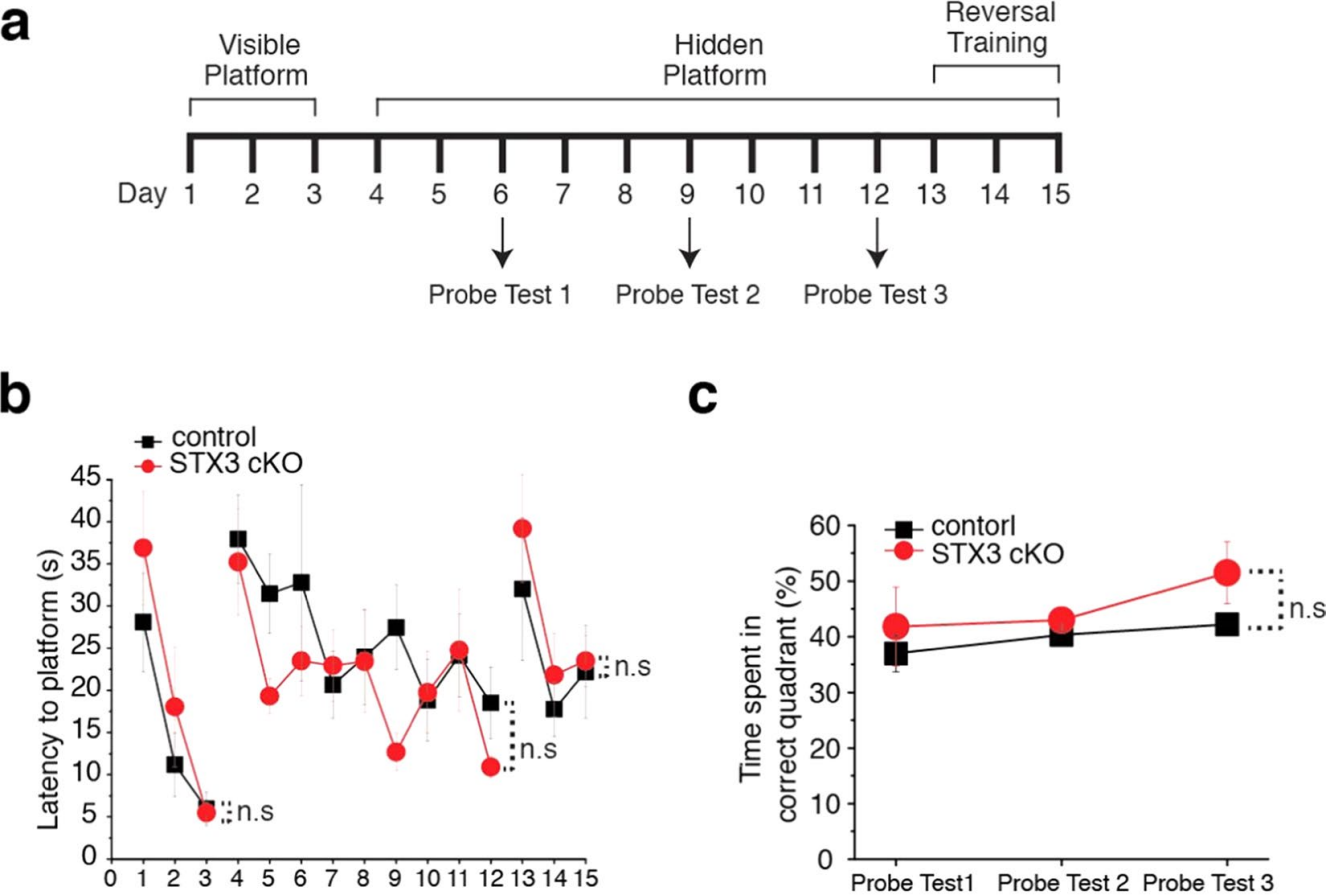
Tissue-specific syntaxin-3 KO mice exhibit normal learning and memory in Morris water maze task. (**a**) Protocol outline used for Morris water maze test. A total of 4 trials were conducted on each animal per day, and entry points to the pool were randomized. The distance and latency to find the platform was measured for a visible platform on days 1 to 3 and a hidden platform on days 4 to 12. The hidden platform location was modified from the visible platform. During the probe test, the platform was removed from the pool and percentage of time spent in the quadrant of the previous location of the platform was calculated from a total recording time of 60 sec. (**b**) The training acquisition curves of finding the visible (days 1 to 3) or hidden (days 4 to 15) platform. Average latency of finding the platform during the 4 daily trials. (**c**) On days 6, 9 and 12, time spent in the quadrant where the platform was previously located was measured. Error bars indicate s.e.m. (animal n = 5 for both groups). Both control and cKO groups spent more time in the trained quadrant than the random 25%. N.S. indicates nonsignificant (p > 0.05) between the two groups; mixed ANOVA test.

**Figure 6:**
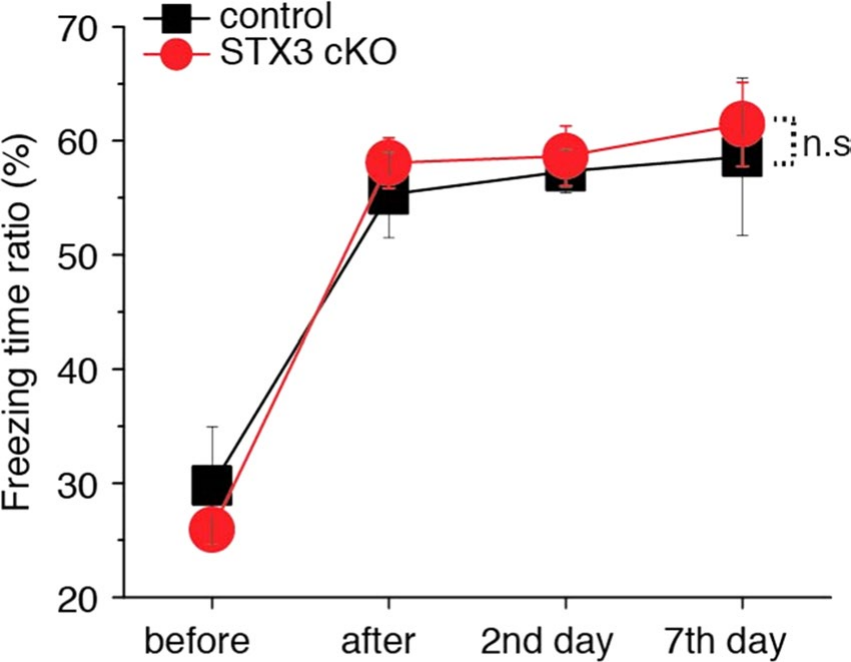
Syntaxin-3 cKO mice exhibit fear conditioning which is indistinguishable from control mice. Freezing time ratio (%) between control (n = 5) and syntaxin-3 cKO (n = 5) mice before shocks, aftershocks, 2nd day post shocks and 7^th^ day post shocks. Control and syntaxin-3 cKO groups exhibited increased freezing behavior after the administration of electrical shock. Both groups retained the increased freezing behavior one and seven days after conditioning. Statistical analyzes found no significant differences (p > 0.05) in freezing behavior of syntaxin-3 flox control mice and syntaxin-3 cKO mice. N.S. indicates nonsignificant; mixed ANOVA test.

To examine whether CaMK2a-Cre mediated deletion of syntaxin-3 from pyramidal neurons affects hippocampal-based learning and memory, we performed two behavioral tasks: Morris water maze (Fig. 5) and contextual fear conditioning (Fig. 6). We implemented the Morris water maze on experimental mice to test their spatial learning and memory^27,28^. For the Morris water maze, mice underwent a total of 15 days in a behavioral protocol and performed 4 trials on each day (Fig. 5a). During each trial, each mouse was placed in a pool of water from a randomized cardinal position. In the syntaxin-3 cKO group (n = 5), the latency to find the visible platform resulted to be similar to that of the control group (n = 5) [mixed ANOVA, F(1,8) = 2.91, p = 0.13] (Fig. 5b). These results indicate that the syntaxin-3 cKO did not impose any functional deficits on the animal’s abilities to visualize their surroundings and to swim. From days 4 to 12, the platform was submerged in water and the mice were assessed to determine if the position of the hidden platform was learned. In both the control and the syntaxin-3 cKO groups, the latency to find the platform on day 4 increased with a subsequent decline during progressive trials and there was no statistically significant difference between the two groups [F(1,8) = 2.39, p = 0.16] (Fig. 5b). From days 13 to 15, the position of the submerged platform was modified (reversal training) and the mice were assessed to determine if the altered position was learned. The latency to find the platform on day 13 increased with a subsequent decline and there was no statistically significant difference between the two groups [F(1,8) = 0.70, p = 0.43] (Fig. 5b). Memory was evaluated by performing a probe test and assessing the time spent in each of the cardinal quadrants. Both the control mice and syntaxin-3 cKO mice spent more time in the previous quadrant that contained the platform. Furthermore, there were no statistical differences in the time spent between the control and cKO groups [F(1,7) = 1.36, p = 0.28] (Fig. 5c) which suggest that learning had occurred, and memory consolidation was normal in cKO mice.

Fear conditioning was performed to further evaluate contextual memory^7,29^ (Fig. 6). Both the cKO and control mice exhibited increases in freezing behavior after shock and freezing behavior was retained 1 and 6 days after initial conditioning. Mixed ANOVA revealed no significant differences in freezing behavior between syntaxin-3 flox control (n = 5) and syntaxin-3 cKO (n = 5) mice [F(1,8) = 0.03, p = 0.87] (Fig. 6). As syntaxin-3 cKO mice do not behaviorally deviate from the syntaxin-3 flox control mice, these results suggest that syntaxin-3 cKO does not cause evident impairment in fear conditioning learning and memory.

## Discussion

SNARE fusion machinery has been postulated to underlie postsynaptic plasticity by regulating the number and composition of neurotransmitter receptors^10–13^. In this study, we examined the role of the t-SNARE syntaxin-3 in synaptic transmission and synaptic plasticity *in vivo* using syntaxin-3 cKO mice (Figs. 1 and 2). We observed no significant differences between syntaxin-3 cKO mice and control mice with respect to basal CA1 neurotransmission, LTP, learning and memory (Figs. 3–6). We also found that the deletion of syntaxin-3 occurs not only in postsynaptic CA1 neurons but also in presynaptic CA3 neurons, but the responses evoked by paired stimulations remained unchanged in cKO mice (Fig. 3). Together these results may argue against a major role of syntaxin-3 in CA1 basal glutamate transmission and LTP.

Our present results are consistent with the previous study that syntaxin-3 is inconsequential for basal transmission (Fig. 3)^11^. However, contrary to the previous finding that suggests an important role of syntaxin-3 during LTP^11^, syntaxin-3 cKO did not cause CA1 LTP impairment in our model (Fig. 4). Furthermore, our results are reflected behaviorally; we found that spatial and contextual learning were intact in syntaxin-3 cKO mice (Figs. 5 and 6) indicating syntaxin-3 is dispensable in hippocampal-based learning. It is possible that the different conclusions may be attributed to the use of different methods to generate models lacking syntaxin-3. Previous work used shRNA mediated silencing to generate syntaxin-3 KDs and although RNAi is an effective tool to reduce the expression of target genes, the potential for off-target and nonspecific effects need to be considered^30^. It is also possible that syntaxin-3 cKO could be developmentally compensated by other syntaxin isoforms, the most likely candidate being syntaxin-4. However, this adaptive compensatory mechanism is triggered by RNA degradation^31^, while the model we selected for deleting syntaxin-3 takes advantage of gene trapping and does not produce unstable mRNA. As such, expression of a stable β-galactosidase/neomycin phosphotransferase fusion (β-geo) mRNA is produced in lieu of syntaxin-3 and RNA instability does not pose a problem in our experiments (Fig. 1). Moreover, the lack of significant changes in syntaxin-3 cKOs cannot be entirely explained by developmental compensation. In our previous study, we showed that syntaxin-4 cKO exhibit significant effects including decreased CA1 fEPSP without changes in presynaptic release probability of the Schaffer collateral-CA1 synapses, reduced magnitude of theta-burst stimulation-induced LTP and impaired spatial learning and memory as demonstrated by Morris water maze test^15^. Therefore, the results obtained from conditional syntaxin-3 and -4 KOs are more consistent with the hypothesis that syntaxin-4 is the critical isoform required for postsynaptic functions.

The discrepancy between our current work and the previous study^11^ regarding LTP may also be explained by the difference in the LTP inducing protocol. In the present experiments, as well as our previous study about the role of syntaxin-4 in LTP^15^, we used a theta-burst stimulation protocol to induce CA1 LTP. This protocol is a widely used and more physiologically relevant LTP inducing protocol believed to be closely related to learning and memory formation^32,33^. Using this protocol, we induced control-like LTP in syntaxin-3 cKO mice (Fig. 4), which were in keeping with unimpaired learning behaviors in Morris Water maze (Fig. 5) and fear conditioning (Fig. 6) tests. On the other hand, the previous study used a stronger high frequency protocol and demonstrated impaired LTP by syntaxin-3 KD^11^. While it remains to be tested whether the LTP induced by the high frequency stimulation is compromised in syntaxin-3 cKO mice, this does not contradict to our present results. Normal theta burst induced LTP by syntaxin-3 cKO mice suggests that AMPARs are inserted to the surface during this type of LTP. Confirming the normal AMPAR receptor trafficking in syntaxin-3 cKO neurons during LTP using more cell biological approaches would be the scope of a future study.

Although syntaxin-4 cKOs strongly reduced basal neurotransmission, it did not completely abolish LTP, suggesting that other syntaxin isoforms such as syntaxin-3 may potentially partially contribute to LTP. To determine whether syntaxin-3 does marginally contribute, it would be necessary to use an experimental method that enables a more acute removal of syntaxin-3 from pyramidal neurons such as tamoxifen-induced conditional knockout. In this case, syntaxin-3 flox mice expressing an inducible CaMK2-Cre gene would be needed. Alternatively, the role of syntaxin-3 could be examined in the background of syntaxin-4 cKO and double flox mice of syntaxin-3 and syntaxin-4 expressing CaMK2a-Cre would need to be generated. With such experiments, it would be possible to determine whether syntaxin-3 does contribute to synaptic plasticity.

Recent studies indicate a distinct vesicular sorting of AMPA and GABA receptors^34^. The use of syntaxin cKO mice in future studies will provide an opportunity to study the role of SNARE proteins not only in the regulation of ionotropic glutamate receptors, but also in GABA receptors since CA1 neurons receive inhibitory inputs from neighboring interneurons in the hippocampus^35^. Such studies will provide new insight into the mechanisms involving the delivery of inhibitory GABA receptors by distinct SNARE proteins.

Syntaxin-3 is an essential protein for survival in mice and is ubiquitously expressed throughout the organism, especially the brain^36^. A recent study using mast cell specific syntaxin-3 KO mice indicated an essential role of this protein in mast cell exocytosis^19^. However, our results suggest that its deletion in CA3 neurons does not impair the release of glutamate (Fig. 3). In contrast, syntaxin-1B has been shown to play a critical role in glutamate release from pyramidal neurons^37^. Therefore, we speculate that syntaxin-3 may play a role in chemical release from other types of neurons or glia. By crossing syntaxin-3 flox mice with other tissue specific Cre mice, the functional role of syntaxin-3 in exocytosis could be elucidated in future experiments. Consistent with the limited impact of syntaxin-3 cKO on hippocampal CA3-CA1 synapses, endogenous syntaxin-3 mRNA expression and its protein level was surprisingly low in hippocampal and cortex pyramidal neurons (Figs. 2 and S2). Future work will elucidate the role of syntaxin-3 in the strongly expressed regions. In summary, our present experiments provide convergent evidence suggesting that syntaxin-3 is dispensable in CA1 basal transmission, LTP and hippocampus-dependent learning and memory.

## Materials and Methods

### Animals

For experiments, we used mice between the ages of 2.5–6 months, without preferences on sex. The mice were maintained in a vivarium on a 12-hr light on/off cycle and at a temperature between 22–23 °C. Mice were given *ad libitum* access to water and food. All experiments performed were in accordance with the guidelines and policies of the Canadian Council on Animal Care and were approved by the animal care committee of the University Health Network.

### Generation of forebrain-specific syntaxin-3 KO mice

Syntaxin-3 flox/flox mice were previously created using embryonic stem (ES) cells from EUCOMM (clone EUCE320f12)^19^. We purchased C57BL/6 mice with CaMK2a-Cre^20^ from the Jackson Laboratory and generated forebrain-specific syntaxin-3 KO mice. Genotyping of mice was performed using PCR by extracting genomic DNA from tail biopsies. Syntaxin-3 conditional KO mice were developmentally normal, fertile and progressed to adulthood without observable behavioral abnormalities. For all experiments, syntaxin-3 flox mice without CaMK2a-Cre were used as the control group.

### X-gal Staining on mouse brain sections

We used a previously described protocol^38^ with slight modifications. The mouse was initially anesthetized using a sodium pentobarbital (Somnotol) (70 mg/kg, intra-peritoneal injection, WTC Pharmaceuticals). The mouse was then perfused with PBS for 5 min and followed by 4% PFA/PBS for another 5 min. The brain was dissected out and post-fixed with 4% PFA/PBS overnight at 4 °C. The fixed brain was washed with PBS and then incubated with 30% sucrose overnight. Sagittal brain slices of 40 µm thickness were obtained via a vibratome (VT1200, Leica Microsystems, Richmond Hill, Canada). These sections were washed three times with PBS and incubated with a staining buffer for 10 min at room temperature. The cryo-section was then incubated with 1 mg/ml X-gal in a staining buffer supplemented with 5 mM Potassium Ferricyanide and 5 mM Potassium Ferrocyanide for 3 hrs at 37 °C.

### Nissl Staining on mouse brain sections

The mouse was initially perfused with PBS for 5 min then subsequently perfused with 10% formalin for an additional 5 min. The mouse brain was dissected out and post-fixed with 10% formalin overnight at 4 °C. The fixed brain was washed with PBS and incubated with 30% sucrose overnight. Cryostat sections of 25 μm were obtained, dried for at least 3 days and then processed with chloroform, 100%, 95%, and 70% ethanol sequentially. Staining with 0.1% cresyl violet was performed at 37 °C for 3 min bath. The sections were then washed quickly in distilled water, differentiated in 90% ethanol for 1–1.5 min and cleared in xylene twice for 5 min.

### *In situ* hybridization

We generated an anti-sense probe with 3′ ~500 bp mouse syntaxin-3 cDNA which was subcloned in PstI-EcoRI site of pBluescript SKII. The plasmids were then linearized by digesting with BamHI and subjected to *in vitro* transcription. *In situ* hybridization on the coronal hippocampus section was carried out as described previously^15^. Briefly, digoxigenin (DIG) labeled anti-sense RNA probe for syntaxin-3 were generated according to manufacture’s protocol (Roche, Laval, Canada). Alkaline phosphatase (AP)-conjugated anti-DIG antibody (Roche) was used to detect hybridized probe. AP activity was detected by 5-bromo-4-chloro- 3-indolyl-phosphate (BCIP, Roche) and 4-nitoro blue tetrazolium chloride (NBT, Roche) in NTMT (100 mM NaCl, 100 mM Tris-Cl pH9.5, 50 mM MgCl2, 0.1% Tween20, 2 mM levamisol).

### Immunofluorescence microscopy

The brain was perfused with PBS and 10% formalin followed by post-fix with 10% formalin overnight at 4 °C. After 30% sucrose incubation, 25 µm sections were sliced using a Leica CM1950 cryostat. Sections were permeabilized with 0.1% TritonX-100 in PBS followed by blocking with 5% bovine serum albumin (BSA) in 0.1% TritonX-100 in PBS. Sections were incubated in anti-Syntaxin-3 antibody (Proteintech, rabbit polyclonal, 1:400). Secondary antibody (Goat anti-Rabbit IgG antibody, Alexa flour 555, Invitrogen) was applied to the sectioned followed by tertiary antibody (Rabbit anti-Goat IgG antibody, Alexa flour 555, Invitrogen) to enhance the signal. Images were taken with a fluorescent microscope (model Olympus BX61).

### Preparation of hippocampal slices for electrophysiological recordings

The animal was anesthetized using sodium pentobarbital as described above. Before decapitation, the animal was infused transcardiacally with an ice-cold high sucrose dissection solution containing (in mM): 300 sucrose, 3.5 KCl, 2 NaH_2_PO_4_, 20 glucose, 0.5 CaCl_2_, 7 MgCl_2_ and 5 HEPES (pH adjusted to 7.4). The brain was rapidly dissected and hemi-sectioned and sagittal slices of 400 µm thickness were obtained via a vibratome. Post sectioning, the brain slices were stabilized in oxygenated (95% O_2_ and 5% CO_2_) artificial cerebrospinal fluid (ACSF) at least 1 hour before recording. The components of ACSF were (in mM): 125 NaCl, 25 NaHCO_3_, 10 glucose, 3.5 KCl, 1.25 NaH_2_PO_4_, 1.3 MgSO_4_, 2 CaCl_2_ (pH7.4 when aerated with 95% O_2_ and 5% CO_2_).

### Electrophysiological recordings

Each brain slice was submerged in a chamber and perfused with oxygenated ACSF at a high flow rate of 15 mL/min. All recordings were conducted at room temperature. Signals were recorded using a 700B amplifier and a digitizer (Digidata 1550, Molecular Devices/Axon Instruments, Sunnyvale, California). Data collection, storage and analysis were completed using PClamp software (version 10, Molecular Devices). These signals were recorded in frequencies between 0–5 kHz and digitized at 50 KHz. To evoke synaptic field potentials, a bipolar stimulating electrode (made of polyimide-insulated stainless-steel wire, outer diameter 0.1 mm; Plastics One, Roanoke, Virginia, USA) was placed in the *stratum radiatum* of the CA2 region. Constant current pulses (10–150 μA) were generated via a Grass stimulator (S88, Natus Neurology Incorporated – Grass Products, Warwick, Rhode Island) and delivered through an isolation unit every 30 sec. Evoked responses were recorded extracellularly from the *stratum radiatum* (apical dendritic layer) of the CA1 region. Recording electrodes were made of thin-walled glass tubes (TW150F-4, World Precision Instruments, Sarasota, Florida) and filled with a solution of 150 mM NaCl and 2 mM HEPES (pH 7.4; resistance of 1–2 MΩ).

For assessing CA1 LTP, field EPSPs (fEPSPs) were evoked at about 30% of the maximal stimulation intensity. Baseline fEPSPs were monitored for 20 min prior to LTP induction. Slices with unstable baseline responses (≥10% variations of baseline response mean) were excluded from additional recordings. A theta burst stimulation with 15 bursts of four pulses at 100 Hz and an inter-burst interval of 200 ms was used to inducing LTP. Following the theta burst stimulation, responses were recorded for an additional 60 min.

### Morris water maze

Mice underwent visible platform training for 3 days and then hidden platform training for 12 days (4 trials per day and inter-trial intervals of 10–15 min). During the hidden platform training, three probe tests were performed at day 3, 6 and 9 and reversal training was conducted at days 10–12. For the visible platform training, if the mice were unable to find the platform within 90 sec, they were guided to the platform by the experimenter’s hand. For the hidden platform training, the procedure was identical to the visible platform training with the exception that the platform was submerged underwater at a depth of 1.5 cm and the platform location was modified. For the reversal training, the platform location was moved to a quadrant different from that in the hidden platform training. The times required to reach the platform during the visible and hidden platform trials and to spend in the pool quadrant in the probe test where the platform was previously located were analyzed.

### Contextual fear conditioning

Mice were placed in a conditioning chamber and allowed to freely explore the chamber for 2.5 min. During the exploring period, freezing behavior (immobility) was measured as a baseline. For fear conditioning, mice experienced 3 rounds of foot shocks (each at 0.75 mA for 2 seconds) and freezing behaviors were assessed during the following 2.5 min. Mice were similarly reassessed 1 and 6 days later to measure contextually conditioned fear memory.

## Supplementary information

Supplementary Figures.
