# Supplementary material for: Syntaxin-3 is dispensable for basal neurotransmission and synaptic plasticity in postsynaptic hippocampal CA1 neurons
Source: Sci Rep. 2020 Jan 20;10:709. doi: 10.1038/s41598-019-57388-6 (PMC6971263; doi:10.1038/s41598-019-57388-6)

**Syntaxin-3 is dispensable for basal neurotransmission and synaptic plasticity in postsynaptic hippocampal CA1 neurons**

Shan Shi^1,2^, Ke Ma^1,2,*^, Na-Ryum Bin^2,3^, Hidekiyo Harada^4^, Xiaoyu Xie^2,5^, Mengjia Huang^2,3^, Haiyu Liu^2,6^, Soomin Lee^2,3^, Xue Fan Wang^3^,^4,^, Roberto Adachi^7^, Philippe P. Monnier^3,4,8^, Liang Zhang^2,9^, and Shuzo Sugita^2,3,*^

Affiliations: ^1^Department of Pediatrics, The First Hospital of Jilin University School of Medicine, Changchun 130021, China; ^2^Division of Fundamental Neurobiology, Krembil Research Institute, University Health Network, Toronto, Ontario, M5T 2S8, Canada; ^3^Department of Physiology, University of Toronto, Toronto, Ontario, M5S 1A8, Canada; ^4^Division of Genetics and Development, Krembil Research Institute, University Health Network, Ontario, M5T 2S8, Canada; ^5^Department of Anesthesiology, Dalian Medical University, Dalian, Liaoning 116044, China; ^6^Department of Neurosurgery, The First Hospital of Jilin University School of Medicine, Changchun 130021, ^7^Department of Pulmonary Medicine, The University of Texas MD Anderson Cancer Center, Houston, Texas 77030; ^8^Department of Ophthalmology & Vision Sciences, ^9^Department of Medicine, Faculty of Medicine, University of Toronto, Toronto, Ontario, M5S 1A8, Canada

*Correspondence: Ke Ma, M.D., Department of Pediatrics, The First Hospital of Jilin University School of Medicine, Changchun 130021, China;

Shuzo Sugita, Ph.D., Krembil Discovery Tower, 7KD-419, University Health Network, 60 Leonard Avenue, Toronto, Ontario, M5T 2S8, Canada

Shuzo.Sugita@uhnresearch.ca

**Supplementary Fig. 1. X-Gal staining to examine the expression of β-galactosidase/neomycin fusion protein in syntaxin-3 cKO mice at three different ages.** (a) X-Gal staining at 8 weeks in syntaxin-3 cKO mice. (b) X-Gal staining at 10 weeks in syntaxin-3 cKO mice. (c) X-Gal staining at 12 weeks in syntaxin-3 cKO mice. Scale bar: 0.5 mm.


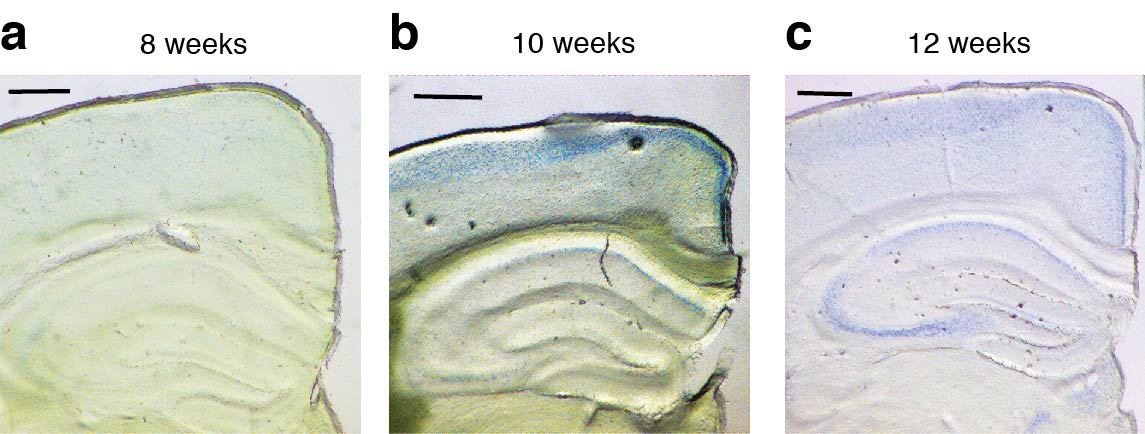


**Supplementary Fig. 2. Syntaxin-3 protein is under the detection level by Immunohistochemistry**. Immunohistochemical staining of Syntaxin-3 on the hippocampus (red). (a) Syntaixn-3 signal is not able to detect on the hippocampus. (b) DAPI staining of hippocampus section (Blue). (c) Merged picture of Syntaxin-3 staining and DAPI. Scale bar: 200 µm


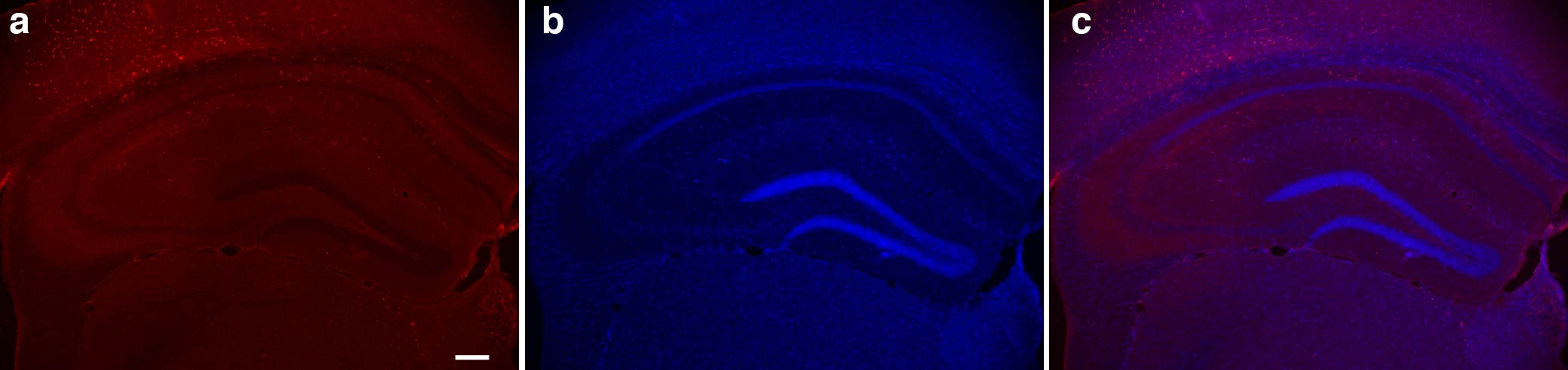

Supplement: Supplementary file 1 — Supplementary Figures. [file 41598_2019_57388_MOESM1_ESM.docx]
